# Cyanine‐Doped Lanthanide Metal–Organic Frameworks for Near‐Infrared II Bioimaging

**DOI:** 10.1002/advs.202104561

**Published:** 2022-01-12

**Authors:** Tao Liang, Zhi Guo, Yifan He, Yanying Wang, Chunya Li, Zhen Li, Zhihong Liu

**Affiliations:** ^1^ Key Laboratory of Analytical Chemistry of the State Ethnic Affairs Commission College of Chemistry and Materials Science South‐Central University for Nationalities Wuhan 430074 China; ^2^ College of Chemistry and Chemical Engineering Hubei University Wuhan 430062 China

**Keywords:** bioimaging, lanthanide, metal–organic frameworks, NIR‐II luminescence, sensitization

## Abstract

Developing metal–organic frameworks (MOFs) with strong near‐infrared II (NIR‐II, 1000–1700 nm) emission is significant for biomedical research but highly challenging. So far there are no MOFs reported for NIR‐II imaging in vivo due to their poor NIR‐II emission efficiency. Herein, a strategy is proposed to prepare MOFs with strong NIR‐II emission, by integrating NIR dye IR‐3C and Ln^3+^ (Ln = Yb, Nd, and Er) into a same framework. IR‐3C with high photon‐absorption ability harvests the excitation photons and transfers energy to Ln^3+^ via a resonance energy transfer pathway, significantly enhancing the NIR‐II emission of Ln^3+^. The as‐obtained Er‐BTC‐IR exhibits excellent NIR‐IIb (1500–1700 nm) emission efficiency in aqueous phase and good biocompatibility after surface modification, which provides advanced bioimaging performance in vivo. It is able to clearly delineate the vessels, spine, and lymph of mice, and also to differentiate the vessels with acute vascular inflammation. This strategy paves the way to the preparation of NIR‐II emissive MOFs and will promote their bioapplication.

## Graphical abstract

Metal–organic frameworks (MOFs) have evoked increasing interest in bioapplicaitions, but their imaging performance is limited by poor photophysical properties. In this work, MOFs with strong NIR‐II emission are fabricated through doping organic NIR dye into Ln‐MOFs (Ln = Yb, Nd, and Er). The obtained Er‐BTC‐IR@A can clearly delineate the vessels, spine, and lymph of mice, and also differentiate acute vascular inflammation.

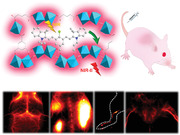

## 1 Introduction

Metal–organic frameworks (MOFs) are an emerging type of porous materials featured with adjustable structures, high porosity, and good biocompatibility, which have been used in a wide variety of areas including catalysis, gas storage and separation, chromatographic, drug delivery, etc.^[^ ^1^ ^]^ Owing to the diversity of metal ions and organic ligands, the photophysical properties of MOFs can be precisely regulated, providing a versatile platform for biosensing and a great potential in in vivo imaging.^[^ ^2^ ^]^ Furthermore, the property of high porosity makes them a kind of ideal material to achieve multimode imaging and fabricate multifunctional platforms.^[^ ^3^ ^]^ However, most of the MOFs are excited by UV or visible photons and emit in visible region, suffering from poor penetration depth, strong autofluorescence, and limited contrast resolution, which has been a long‐term bottleneck to restrict their bioapplication.^[^ ^4^ ^]^

Near‐infrared II (NIR‐II, 1000–1700 nm) imaging, especially in the NIR‐IIb (1500–1700 nm) region, has evoked increasing attention in the recent years due to its significantly higher penetration and resolution originating from the notably decreased scattering and absorption in this region.^[^ ^5^ ^]^ Therefore, it is of great significance to develop MOFs with strong NIR‐II emission and hence suitable for use in vivo. Rational design of ligands is an effective way to gain redshift of the emission wavelength.^[^ ^6^ ^]^ However, it needs a complex synthesis and modification process, and moreover, the emission peaks of the developed organic dyes can hardly reach to NIR‐IIb region.^[^ ^7^ ^]^ Another approach to endow MOFs with NIR‐II emission is to employ lanthanide ions (Ln^3+^) as metal nodes, which possess abundant 4f electron orbital levels and multiple emission in NIR‐II region. Ln‐MOFs (Ln = Yb^3+^, Nd^3+^, and Er^3+^) have been recognized as a kind of NIR‐II luminophors.^[^ ^8^ ^]^ Unfortunately, because of the extremely faint photon‐absorption ability of Ln^3+^, Ln‐MOFs are subject to rather low NIR‐II emission efficiency.^[^ ^9^ ^]^ Despite the great effort made in recent years,^[^ ^10^ ^]^ no MOFs have been actually applied for NIR‐II imaging in vivo so far.

**Scheme 1:**
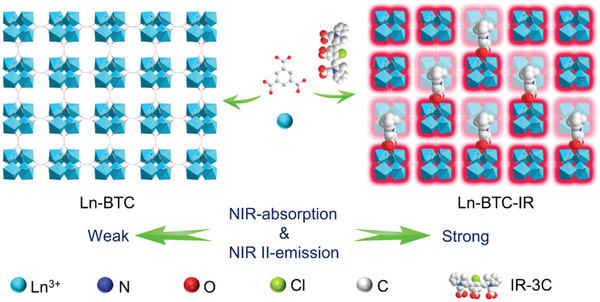
Schematic illustration of fabricating Ln‐MOFs with strong NIR‐II emission through cyanine dye‐doping process.

Herein, we aim to develop Ln‐MOFs with strong NIR‐II/IIb emission which can be competent for in vivo imaging. It is known that organic dyes are able to serve as “antenna” to absorb photons and transfer their excited‐state energy to metal ions.^[^ ^11^ ^]^ Thus, we integrated cyanine, an NIR dye with strong NIR–photon absorption ability and high emission efficiency, with Ln^3+^ into a same framework to sensitize the NIR‐II emission of Ln‐MOFs. We adopted the doping strategy instead of tagging the dye on the MOFs surface or directly employing the dye as the ligand mainly for two reasons: 1) doping the dye molecules into the crystal lattice of MOFs can effectively shorten the distance between “antenna” and Ln^3+^, ensuring sufficient energy transfer efficiency,^[^ ^12^ ^]^ and 2) doping is a less rigorous approach which helps to simplify the structures of the dye and prevent their decomposition during preparation of MOFs. Following this conception, we used IR‐3C, a cyanine dye with three carboxyl groups as binding groups, as the “antenna” and doped it into Ln‐BTC (BTC = 1,3,5‐benzenetricarboxylate as ligand, Ln = Yb, Nd, and Er) to achieve Ln‐MOFs with superior NIR‐II emission performance (**Scheme** **1**). Subsequently, by coating with amphiphilic molecules, phosphatidylcholine (PC) and DSPE–PEG–COOH, the as‐obtained Ln‐BTC‐IR@A showed great water‐dispersibility, which is beneficial to bioapplication. Especially, Er‐BTC‐IR@A with emission peak in NIR‐IIb region exhibited much superior bioimaging performance, which can not only distinctly depict the structures of vessels, spine, and lymph of mice, but also successfully monitor the change of vessel structures and hemodynamics in acute vascular inflammation.

## 2 Results and Discussion

### 2.1 Synthesis and Characterization of Er‐BTC‐IR

**Figure 1:**
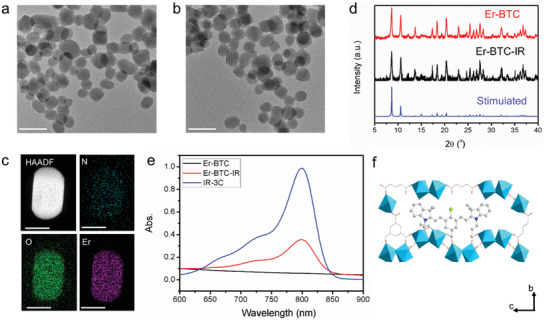
TEM images of a) Er‐BTC and b) Er‐BTC‐IR. c) HAADF image and corresponding elemental mapping of Er‐BTC‐IR. d) XRD patterns of Er‐BTC and Er‐BTC‐IR. The standard pattern was simulated according to the X‐ray single crystal diffraction in the previous report.^[^ ^13^ ^]^ e) Absorption spectra of IR‐3C, Er‐BTC, and Er‐BTC‐IR. f) Schematic illustration of the possible structure of Er‐BTC‐IR. Scar bars in (a), (b), and (c) are 100 nm, 100 nm, and 50 nm, respectively.

To verify the feasibility of our strategy, IR‐3C was first doped into Er‐BTC to boost the NIR‐IIb emission arising from ^4^I_13/2_→^4^I_15/2_ transition of Er^3+^. The obtained Er‐BTC‐IR were spherical particles with an average diameter of ≈50 nm as shown in the transmission electron microscopy (TEM) image, which was similar to that of Er‐BTC (**Figure** **1**a,b). High‐angle annular dark field (HAADF) image and corresponding elemental mapping illustrated that Er, N, and O uniformly distributed in the particles (Figure 1c). The high crystallization was verified by the sharp X‐ray powder diffraction (XRD) peaks, which were in accordance with the previous report.^[^ ^13^ ^]^ The XRD pattern of Er‐BTC‐IR was consistent with Er‐BTC, demonstrating that doping IR‐3C did not deteriorate the crystal walls (Figure 1d). The absorption spectrum of Er‐BTC‐IR displayed a strong absorption peak around 799 nm originating from IR‐3C, which confirmed the successful doping of IR‐3C in Er‐BTC (Figure 1e). Though the size of IR‐3C (2.1 nm × 0.8 nm, as shown in Figure S1, Supporting Information) is slightly larger than the pore of Ln‐BTC (≈5.8 × 5.8 Å^2^),^[^ ^13^ ^]^ it can be successfully encapsulated through a reported one‐pot synthesis method.^[^ ^14^ ^]^ To clearly elucidate the interaction between IR‐3C and Er‐BTC, IR‐2C and IR‐0C with two and no carboxyl groups, respectively, were synthesized as control dyes and doped into Er‐BTC. The doping efficiency (the ratio of dye contents doped into MOFs to the added amounts of dye) of these dyes was estimated according to the absorbance and extinction coefficient (Figures S2 and S3a–c, Supporting Information). As shown in Figure S3d of the Supporting Information, the doping efficiencies were 31%, 11%, and 0% for IR‐3C, IR‐2C, and IR‐0C, respectively, indicating that the cyanine dyes combined with Er‐BTC through the coordination between carboxyl groups and Er^3+^ and all of the three carboxyl groups of IR‐3C formed coordination bonds with Er^3+^. The possible structure of Er‐BTC‐IR was displayed in Figure 1f.

### 2.2 IR‐3C‐Sensitized NIR‐II Luminescence of Er‐BTC

**Figure 2:**
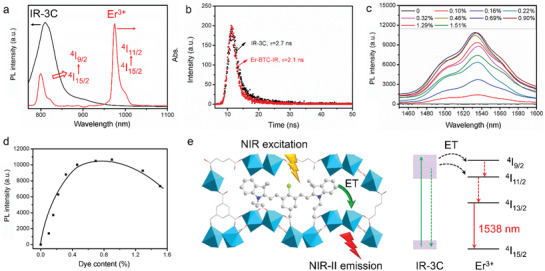
a) Normalized emission spectrum of IR‐3C and the absorption spectrum of Er^3+^. b) Time‐resolved luminescence spectra of IR‐3C dissolved in DMSO and doped in Er‐BTC. c) NIR‐IIb emission spectra and d) the intensities at 1538 nm of Er‐BTC‐IR with doping different contents of IR‐3C in DMSO. e) Schematic illustration of the energy transfer cascade in Er‐BTC‐IR excited by 808 nm laser. The dotted arrows and solid arrows represent nonradiative transition and radiative transition, respectively. “ET” is the abbreviation of “Energy Transfer”.

To achieve NIR‐II luminescence enhancement after IR‐3C doping, the effective energy transfer from IR‐3C to Ln^3+^ and improved photon‐absorption ability of Ln^3+^ are the two prerequisites. As shown in **Figure** **2**a, IR‐3C displayed strong emission in the range from 800 to 1100 nm, which overlapped with the absorption of Er^3+^ around 799 nm (^4^I_15/2_→^4^I_9/2_) and 974 nm (^4^I_15/2_→^4^I_11/2_), ensuring the occurrence of resonance energy transfer process. After doping into Er‐BTC, the lifetime of IR‐3C dropped from 1.2 to 1.0 ns (Figure 2b), indicating an energy transfer efficiency of 22% as calculated by (1‐*τ*_MOF_/*τ*_free_) × 100%, where *τ*_free_ and *τ*_MOF_ are the luminescence lifetimes of IR‐3C dissolved in DMSO and doped in Er‐BTC, respectively. As shown in Figure S2a,b of the Supporting Information, IR‐3C possessed a high molar absorption coefficient of 8.8 × 10^4^ m ^−1^ cm at 808 nm (the wavelength of generally commercial laser for NIR‐II imaging), which is 6.9 × 10^5^‐fold higher than that of Er^3+^ (0.13 m ^−1^·cm, Figure S4, Supporting Information). Above results strongly suggested that IR‐3C fulfilled all of the requirements for sensitizing the NIR‐IIb emission of Er‐BTC. As expected, Er‐BTC‐IR emitted strong NIR‐IIb luminescence, while no obvious NIR‐IIb emission of Er‐BTC was measured under the same detection condition (Figure 2c). As the amount of doped IR‐3C increasing, NIR‐IIb luminescence intensity of Er‐BTC‐IR enhanced first and reached the maximum when the IR‐3C content was 0.9% (the mass ratio of IR‐3C to Er‐TBC‐IR, Figure 2d; Figure S5, Supporting Information). The possible NIR‐IIb luminescence mechanism of Er‐BTC‐IR was depicted in Figure 2e. Briefly, IR‐3C absorbed the energy of excitation photons to reach the excited state, and then transferred this energy to Er^3+^ to generate more excited ions populated in the ^4^I_9/2_ and ^4^I_11/2_, emitting the elevated NIR‐IIb emission. Whereas, an exorbitant amount of IR‐3C induced the decrease of NIR‐IIb emission, maybe because of the slight aggregation caused quenching effect of IR‐3C, which is a common phenomenon occurring at high dye concentrations.

### 2.3 Versatility of the Strategy to Enhance NIR‐II Emission of Ln‐MOFs

**Figure 3:**
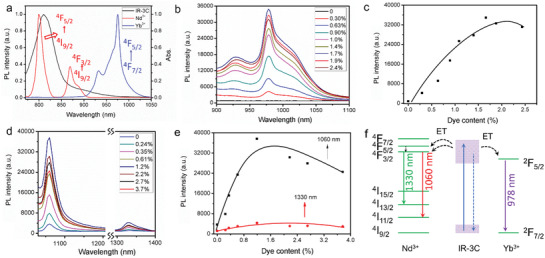
a) The spectral overlap between the emission spectrum of IR‐3C and the absorption spectra of Nd^3+^ and Yb^3+^. The NIR‐II emission spectra of b) Yb‐BTC‐IR and d) Nd‐BTC‐IR and the corresponding luminescence intensities of the emission peaks c,e) with doping various contents of IR‐3C in DMSO. f) Schematic illustration of energy transfer cascade in Yb‐BTC‐IR and Nd‐BTC‐IR excited by 808‐nm laser.

As shown in **Figure** **3**a, the emission of IR‐3C also overlapped with other generally used NIR‐II emissive Ln^3+^ ions, i.e., Yb^3+^ and Nd^3+^, thus we looked into the possibility of enhancing luminescence of other Ln‐MOFs via the above described energy cascade with this dye. To this end, Yb‐BTC‐IR and Nd‐BTC‐IR were fabricated by the same approach with Er‐BTC‐IR. The XRD patterns, TEM, elemental mapping, and absorption spectra confirmed the successful synthesis of these two materials (Figures S6–S8, Supporting Information). As expected, Yb‐BTC was almost nonemissive, but Yb‐BTC‐IR emitted strong luminescence ≈980 nm arising from ^4^F_5/2_→^4^F_7/2_ transition of Yb^3+^ (Figure 3b,c). IR‐3C can also sensitize the NIR‐II emission of Nd‐BTC. Different from Yb^3+^ and Er^3+^, Nd^3+^ possesses two emission peaks in NIR‐II region, which are assigned to ^4^F_3/2_→^4^I_11/2_ transition (1060 nm) and ^4^F_3/2_→^4^I_13/2_ transition (1330 nm). Because both of the two emission bands originate from the radiative transition of Nd^3+^ in ^4^F_3/2_ energy state, which can be sensitized by IR‐3C, they are enhanced simultaneously after IR‐3C doping (Figure 3d,e). The energy transfer pathways in Yb‐BTC‐IR and Nd‐BTC‐IR were depicted in Figure 3f.

### 2.4 Surface Modification of Er‐BTC‐IR

**Figure 4:**
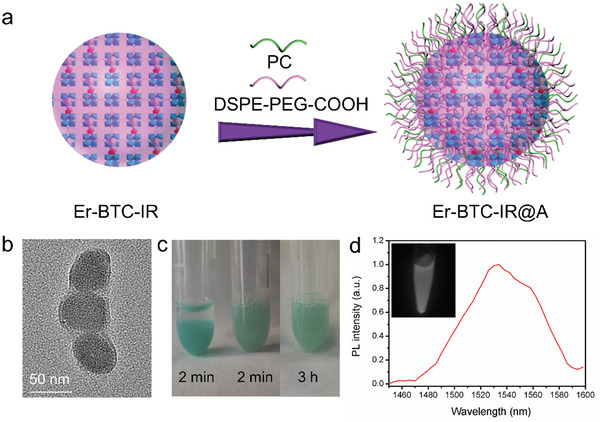
a) Schematic illustrations of amphiphilic molecules coating on the surface of Er‐BTC‐IR to obtain the water‐dispersible materials Er‐BTC‐IR@A. b) TEM image of Er‐BTC‐IR@A. c) Photograph of Er‐BTC‐IR (left) and Er‐BTC‐IR@A (middle and right) after being dispersed in water for different time. d) NIR‐IIb luminescence image and luminescence spectra of Er‐BTC‐IR in aqueous phase excited by 808‐nm laser.

Er‐BTC‐IR was chosen for subsequent investigation on the bioimaging performance of dyes‐doped Ln‐MOFs, in consideration of its superior property of NIR‐IIb emission. Er‐BTC‐IR was first transferred into water‐dispersible materials to facilitate the bioapplication. Amphiphilic molecules, DSPE–PEG–COOH and PC (*m*/*m* = 1/1), were coated on the surface of Er‐BTC‐IR to obtain the dispersibility in aqueous phase (**Figure** **4**a). The layer of amphiphilic molecules can be clearly observed in the TEM image (Figure 4b). The increased hydrodynamic diameter of Er‐BTC‐IR also verified the successful modification of DPES–PEG–COOH and PC, which increased from 220 to 295 nm (Figure S9, Supporting Information). As shown in Figure 4c, Er‐BTC‐IR deposited within 2 min in an aqueous phase, while it can be dispersible for hours after coating with amphiphilic molecules. Significantly, Er‐BTC‐IR@A exhibited sufficient emission efficiency in the aqueous phase (Figure 4d), which fulfilled the prerequisite for bioimaging. The control material, Er‐BTC coated with DPES–PEG–COOH and PC, was nearly nonemissive in NIR‐IIb region (Figure S10, Supporting Information), which demonstrated that the NIR‐IIb luminescence enhancement of Ln‐MOF was essentially contributed by IR‐3C doping rather than the amphiphilic molecules coating. NIR‐IIb emission intensity of Er‐BTC‐IR@A kept stable within 5 min under the exposure of 808 nm laser and changed slightly in aqueous buffer solutions with various pH (4.9–7.9, Figure S11, Supporting Information), providing adequate stability for bioimaging.

### 2.5 NIR‐IIb Luminescence Imaging Performance in Living Mouse

Before application in bioimaging, biocompatibility and biosafety of Er‐BTC‐IR@A were first investigated. Methyl thiazolyl tetrazolium (MTT) assay showed that the cell viability of HeLa cells remained almost 100% after incubating with different concentrations (0–0.5 mg mL^−1^) of Er‐BTC‐IR@A for 24 h, indicating the low toxicity of Er‐BTC‐IR@A (Figure S12, Supporting Information). This result was further proved by costaining U87 cells with PI and Calcein‐AM, which can selectively lighten up dead cells and living cells, respectively (Figure S13, Supporting Information). No obvious fluorescence from PI was detected, thus confirming the high cell viability. Blood biochemistry analysis, blood routine analysis, and H&E staining of major organs were conducted with healthy Kunming mice. After intravenous (i.v.) injection of Er‐BTC‐IR@A, the mice were normally fed for 10 and 20 days and then were sacrificed and the blood and major organs (heart, liver, spleen, lung, and kidney) were harvested. As shown in Figures S14 and S15 of the Supporting Information, the blood analysis and H&E staining of Er‐BTC‐IR@A treated mice displayed no significant difference with the mice in the control group, which demonstrated this material was safe enough for bioapplications.

**Figure 5:**
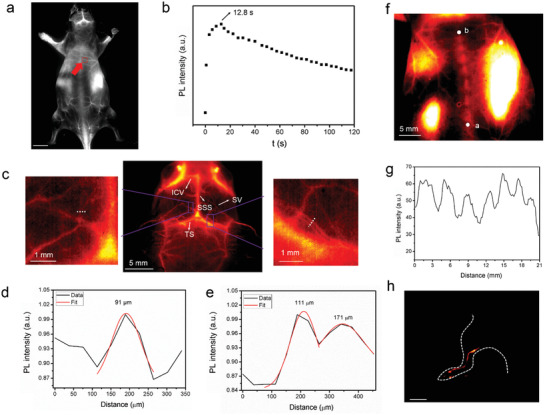
a) The NIR‐II luminescence image of Kunming mouse after injection of Er‐BTC‐IR@A for 12.8 s. b) The time course of luminescence intensities of the vessel pointed by the red arrow in (a). c) Cerebral vessels images of mice after injection of Er‐BTC‐IR@A. The cross‐sectional intensity profiles, Gaussian functional fitting lines and FWHM values along the white dotted line in the d) left image and e) right image of (c). f) The NIR‐II luminescence image of the spine. g) Cross‐sectional intensity profiles from point “a” to point “b” in (f). h) The NIR‐II luminescence image of lymph in the leg of Kunming mouse (the outline of the mouse was depicted by the white dotted line) 1 min after injection of Er‐BTC‐IR@A. The scar bar in (a) and (h) is 1 cm.

The imaging performance at different emission wavelengths was compared to illustrate the advantage of NIR‐II/IIb imaging. The capillary filled with different luminophors was covered by 1% intralipid to simulate the physiological environment and the luminescence images were acquired. As shown in Figure S16 of the Supporting Information, NIR‐II/IIb luminescence images were obviously clearer than the images in visible region. By using the cross‐sectional luminescence intensity analysis and Gaussian fits method, the full width at half‐maximums (FWHM) of capillary were 5.6, 4.5, and 4.1 mm in visible region image, NIR‐II image and NIR‐IIb image, respectively, which indicated the improved imaging performance in NIR‐II region, especially NIR‐IIb region because of the decreased photon‐scattering as well as elevated resolution. Subsequently, Er‐BTC‐IR@A with NIR‐IIb emission was i.v. injected into Kunming mice to investigate the imaging performance in vivo. As shown in **Figure** **5**a, the vessels of the whole body can be clearly depicted. The NIR‐II luminescence signal in vessels reached the maximum at 12.8 s postinjection (p.i.), then gradually decreased and enriched into liver, spleen, etc. (Figure 5b; Figures S17 and S18, Supporting Information). NIR‐IIb luminescence imaging of cerebrovascular structure, such as superior sagittal sinus, transverse sinus, inferior cerebral veins, and superficial veins can also be obtained in spite of the block of cranial bones. Tinny vessels were distinctly observed as shown in Figure 5c. By drawing NIR‐II luminescence intensity profiles across the tinny vessel and fitting with Gaussian functions, the FWHM value was calculated as low as 91 µm (Figure 5d). Two adjacent tiny vessels can also be easily distinguished (Figure 5c,e), which confirmed the high spatial resolution of NIR‐IIb luminescence imaging. By the NIR‐IIb imaging with Er‐BTC‐IR@A, we found the spine was lightened up because of the high binding ability of Ln^3+^ with hydroxyapatite, which is a main component of spine (Figure 5f).^[^ ^15^ ^]^ The NIR‐II emission almost vanished 20 min after injection, indicating the fast metabolism from spine (Figure S19, Supporting Information). Benefitting from the striking penetration depth and imaging resolution, different vertebras were unambiguously delineated (Figure 5g). In addition, Er‐BTC‐IR@A was intradermally injected into the rear paw of Kunming mouse to investigate its capability to visualize the lymph. Notably, popliteal and sciatic lymph was able to be clearly identified within 1 min p.i. (Figure 5h). Apart from Er‐BTC‐IR@A, Nd‐BTC‐IR@A and Yb‐BTC‐IR@A also can be applied to visualize the vessels and spine structure of Kunming mouse (Figure S20, Supporting Information). Due to the relatively shorter emission wavelength and higher photon‐scattering, images acquired with Nd‐BTC‐IR@A and Yb‐BTC‐IR@A were not as clear as that acquired with Er‐BTC‐IR@A. Nonetheless, they still showed much better imaging performance compared with fluorophores with visible emission (Figure S21, Supporting Information), by which tinny structures, such as vessels, cannot be differentiated.

### 2.6 NIR‐IIb Luminescence Imaging in Mice with Acute Vascular Inflammation

**Figure 6:**
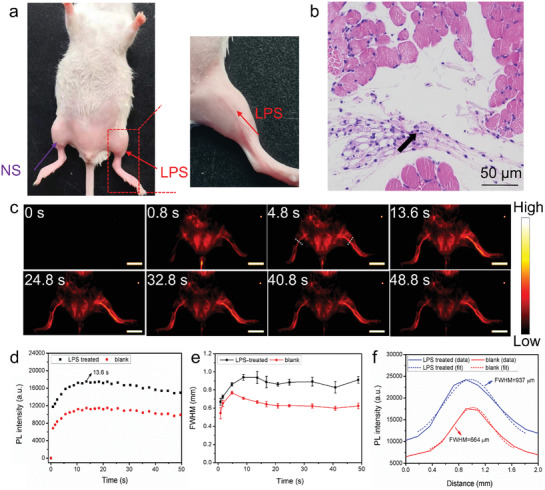
a) Photographs showing the injection sites of LPS and physiological saline (NS). b) H&E staining of the right hindlimb after LPS injection for 4 h. Lymphocytic infiltrate was pointed by the black arrow. c) NIR‐II vessel images of acute vascular inflammation at different time points after Er‐BTC‐IR@A injection. Scale bar: 1 cm. The time course of d) luminescence intensity and e) FWHM of the vessels pointed by the white dotted lines in (c). Data were shown as Mean ± SD of three animals. f) Cross‐sectional intensity profiles, Gaussian functional fitting lines, and FWHM values of the vessels pointed by the white dotted lines in (c) at 13.6 s after injection of Er‐BTC‐IR@A.

Encouraged by the excellent imaging performance of Er‐BTC‐IR@A, we tried to apply the Ln‐MOFs in monitoring real physiological processes. It is well known that the incidence of acute vascular inflammation can induce the change of vessel structures and hemodynamics, such as vasodilation and the increased blood flow. Thus, we expected it can be monitored in real time by NIR‐IIb luminescence imaging with Er‐BTC‐IR@A. Lipopolysaccharide (LPS) was subcutaneously injected into the right hindlimb of mice to induce vascular inflammation and the left hindlimb was injected with physiological saline as the control group (**Figure** **6**a). The occurrence of inflammation was verified by the lymphocytic infiltrate observed in the H&E staining image of right hindlimb (Figure 6b), which was not observed in that of the left hindlimb (Figure S22, Supporting Information). Er‐BTC‐IR@A was then i.v. injected into the mice and the vessels can be rapidly visualized within several seconds p.i. (Figure 6c). Significantly, NIR‐IIb luminescence intensity of the vessel in the right hindlimb (LPS treated) was obviously higher than that of the left hindlimb (physiological saline treated), confirming the increased blood flow in the acute vascular inflammation (Figure 6c,d). Furthermore, the increased diameter of vessels was distinctly observed. As shown in Figure 6e,f, the FWHM of the vessel of the right hindlimb was ≈270 µm broader than that of the left hindlimb. By contrast, the luminescence intensity of vessels in different hindlimbs without LPS treatment displayed no obvious difference (Figure S23a,b, Supporting Information). Besides, FWHM of the vessels fluctuated between 560 and 680 µm and the difference of these vessels’ FWHM was less than 60 µm after injection for 13.6 s (Figure S23c,d, Supporting Information). Above results convincingly demonstrated that Er‐BTC‐IR@A can rapidly differentiate the vessel with acute inflammation through a noninvasive manner, which may provide a powerful method for acute vascular inflammation diagnosis.

## 3 Conclusion

In this work, we put forward a strategy to obtain NIR‐II/IIb emissive MOFs with high luminescence efficiency, which are available for bioimaging. NIR organic dye, IR‐3C, was doped into Ln‐MOFs (Ln = Yb^3+^, Nd^3+^, and Er^3+^) to increase the ability of absorbing excitation photons, thus achieving the significant improvement of NIR‐II emission. The obtained Er‐BTC‐IR@A with surface modification showed strong NIR‐IIb emission in aqueous phase and possessed excellent biocompatibility, which was suitable for application in the biological environment. NIR‐IIb luminescence imaging of vessels, spine, and lymph with high resolution was successfully achieved after i.v. injection of Er‐BTC‐IR@A into mice. Benefitting from the superior imaging performance of Er‐BTC‐IR@A, vasodilation and blood flow increase were observed in acute vascular inflammation process, which may provide a powerful tool for diagnosis of acute vascular inflammation. We believe that this study will inspire the development of MOFs in NIR‐II imaging and broaden their application in the biomedical field.

## 4 Experimental Section

### Materials and Instruments

DSPE‐PEG‐COOH was purchased from Xi'an ruixi Biological Technology Co., Ltd. 3‐iodopropionic acid, 4‐oxocyclohexanecarboxylic acid, and 2,3,3‐trimethylindolenine were obtained from Aladdin Reagent, Ltd. (Shanghai, China). Other used chemical reagents were also purchased from Sinopharm Chemical Reagent Co., Ltd. (Shanghai, China). All chemical reagents were analytical grade or higher and used without further purification. Kunming mice were supplied by China three gorges university (Yichang, China) and received care in compliance with the guidelines outlined in the Guide for the Care and Use of Laboratory Animals and the procedures were approved by the Animal Care and Use Committee of South‐Central University for Nationalities.

TEM images were obtained from Talos F200X transmission electron microscope. XRD patterns were measured by the Escalab 250Xi (Thermo, USA). The emission spectra and time‐resolved luminescence spectra were measured by FLS1000 spectrometer (Edinburgh Instrument) equipped with 808 nm laser or 650 nm laser. The absorption spectra were acquired by a UH‐4150 spectrophotometer (Hitachi, Japan). MTT test were recorded by a Multiskan GO microplate reader (Thermo, USA). NIR‐II in vivo luminescence imaging was conducted on the In‐Vivo Master NIR‐II luminescence imaging system (Gand Imaging Technology Co. Ltd., Wuhan) equipped with 808 nm laser.

### Synthesis of IR‐2C and IR‐0C

IR‐2C was synthesized according to the literature.^[^ ^16^ ^]^

### Synthesis of IR‐3C

The synthetic route was depicted in Scheme S1 of the Supporting Information.

Compound **3**. Compound 3 was synthesized according to the literature.^[^ ^16^ ^]^ Briefly, 1.69 g of 2,3,3‐trimethylindolenine (**1**, 10 mmol) and 2 g of 3‐iodopropionic acid (**2**, 10 mmol) were added into 20 mL of toluene. The solution was refluxed overnight. After cooling down to r.t., compound **3** was obtained by filtration and washed by EA as a light pink solid (2.87 g, 80% yield).

Compound **5**. 10 mL of anhydrous DMF was added into a round‐bottom flask and stirred at 0 °C for 10 min. 2.54 mL of POCl_3_ (26.5 mmol) was added dropwise and kept reaction for 30 min. Then, 1.5 g of 4‐oxocyclohexanecarboxylic acid (**4**, 10.6 mmol) in DMF was added slowly and the solution was heated to 80 °C with stirring overnight. After cooling down to r.t., the solution was pooled into 200 mL of ice water. Compound **5** precipitated after several hours and was collected by filtration, then was dried under vacuum and stored at −20 °C (1.2 g, 53% yield). It was used for next reaction without purification for instability. ^1^H NMR (400 MHz, DMSO) *δ* 12.28 (s, 1H), 11.10 (s, 1H), 8.82 (s, 1H), 2.73 (dd, J = 15.7, 3.6 Hz, 2H), 2.56 (m, 1H), 2.37 (dd, J = 15.9, 9.4 Hz, 2H). HRMS (m/z): [M‐H]^−^ calcd for [C_9_H_9_ClO_4_] 215.0117, found 215.0131 (Figure S24, Supporting Information).

IR‐3C. 302 mg of compound **5** (1.4 mmol), 1 g of compound **3** (2.8 mmol), and 459 mg of sodium acetate (5.6 mmol) were added into 10 mL of acetic anhydride. The mixture was stirred at r.t. for 2 h. It was then poured into 200 mL of water and the supernatant was discarded. The residue was purified by silica gel column chromatography with CH_2_Cl_2_/MeOH/AcOH as eluent. The eluent was removed under vacuum to obtain IR‐3C as a dark green solid (270 mg, 30% yield). ^1^H NMR (400 MHz, DMSO) *δ* 12.57 (s, 3H), 8.27 (d, J = 14.1 Hz, 2H), 7.64 (d, J = 7.4 Hz, 2H), 7.45 (m, 4H), 7.34‐7.25 (td, J = 7.4, 1.4 Hz, 2H), 6.46 (d, J = 14.2 Hz, 2H), 4.47 (t, J = 6.8 Hz, 4H), 3.09 (d, J = 12.1 Hz, 2H), 2.82‐2.66 (m, 7H), 1.67 (d, J = 1.2 Hz, 12H) (Figure S25, Supporting Information). ^13^C NMR (101 MHz, DMSO) *δ* 175.75, 173.20, 172.53, 147.70, 143.81, 142.20, 141.57, 129.08, 125.75, 124.89, 122.98, 112.14, 102.62, 49.57, 37.65, 32.10, 28.62, 28.00, 27.92 (Figure S26, Supporting Information). [M‐2H]^−^ calcd for [C_37_H_38_ClN_2_O_6_] 641.2424, found 641.2388 (Figure S27, Supporting Information).

### Synthesis of Ln‐BTC

Ln‐BTC were synthesized based on the previous report.^[^ ^17^ ^]^ To a solution consisted of BTC (1 mL, 40 × 10^−3^ m in DMF), LnCl_3_·6H_2_O (1 mL, 80 × 10^−3^ m in DMF), and H_2_O (0.2 mL), 0.3 mL of NaAc (400 × 10^−3^ m in H_2_O) was added dropwise at r.t. under stirring and kept reaction for 10 min. The mixture was then heated to 60 °C and kept undisturbed for 30 min. Ln‐BTC was obtained by centrifugation for 15 min and washed with DMF for twice. About 10–20 mg white solid can be acquired after drying under vacuum.

### Synthesis of Ln‐BTC‐IR

The synthesis procedure is similar to that of Ln‐BTC, except for the presence of IR‐3C before adding NaAc.

### Synthesis of Er‐BTC‐IR@A

25 µL of PC (a.q., 100 mg mL^−1^), 25 µL of DSPE‐PEG‐COOH (a.q., 100 mg mL^−1^) and 1 mL of ultrapure water were added into 5 mg of Er‐BTC‐IR in 2 mL centrifugation tube. The mixture was dispersed under ultrasound for about 1 min and then centrifuged for 15 min. Er‐BTC‐IR@A was obtained after washed by ultrapure water and dispersed in ultrapure water.

### MTT Test

Various concentrations (0–0.5 mg mL^−1^) of Er‐BTC‐IR@A were incubated with HeLa cells in fresh DMEM medium (each concentration was conducted in six replicates). The cells were then cultured for 24 h at 37 °C under 5% CO_2_. MTT reagent was added with a final concentration of 1 mg mL^−1^ and the cells were incubated for another 4 h. DMSO was then added to dissolve formazan produced by living cells. The absorbance at 490 nm was measured to calculate the survival rate of HeLa cells (cell viability = Mean Abs. of Er‐BTC‐IR@A treated wells/mean Abs. of control wells) × 100%).

### In Vivo Biosafety Assessment

Kunming mice (female, ≈30 g) were intravenously (i.v.) injected with 100 µL of Er‐BTC‐IR@A (20 mg mL^−1^) or normal saline. Each group contained five mice. After fed for 10 days and 20 days, the mice were sacrificed to collect blood and the major organs (heart, liver, spleen, lung, and kidney) for blood biochemistry and blood routine analysis as well as H&E staining.

### NIR‐IIb Imaging of Vessels and Spine

100 µL of 5% chloral hydrate was intraperitoneally injected into Kunming mice (female, ≈30 g). The fur of the mice was removed by depilatory cream. Then, 100 µL of Er‐BTC‐IR@A (20 mg mL^−1^) was i.v. injected into Kunming mouse. NIR‐IIb images were recorded by in vivo NIR‐II imaging system equipped with an 808 nm laser (80 mW cm^−2^) and 1300 nm LP optical filter.

### NIR‐IIb Imaging of Lymph

25 µL of Er‐BTC‐IR@A (20 mg mL^−1^) was subcutaneously injected into the rear paw of fur‐removed Kunming mice. After 1 min, the NIR‐II image was obtained by the in vivo NIR‐II imaging system equipped with an 808 nm laser (80 mW cm^−2^) and 1300 nm LP optical filter.

### NIR‐IIb Vessel Imaging of Mice with Acute Vascular Inflammation

25 µL of LPS (10 mg mL^−1^) was subcutaneously injected into the right hindlimb of fur‐removed mice to cause acute vascular inflammation. 25 µL of normal saline was injected into the left hindlimb to serve as a control. After 4 h, 100 µL of Er‐BTC‐IR@A (20 mg mL^−1^) was i.v. injected into the mouse and NIR‐IIb imaging was recorded immediately by the in vivo NIR‐II imaging system equipped with an 808 nm laser (80 mW cm^−2^) and 1300 nm LP optical filter.

### Statistics

All experiments were independently repeated for at least three times. Data are given as Mean ± SD. ImageJ was the software for bioimaging analysis, the others was conducted by Origin.

## Conflict of Interest

The authors declare no conflict of interest.

## Supporting information

Supporting Information

## Data Availability

The data that support the findings of this study are available from the corresponding author upon reasonable request.
