# Supplementary material for: Cyanine‐Doped Lanthanide Metal–Organic Frameworks for Near‐Infrared II Bioimaging
Source: Adv Sci (Weinh). 2022 Jan 12;9(7):2104561. doi: 10.1002/advs.202104561 (PMC8895151; doi:10.1002/advs.202104561)
Supplement: Supplementary file 1 — Supporting Information [file ADVS-9-2104561-s001.pdf]

WILEY-VCH

ADVANCED SCIENCE
Open Access

Supporting Information

for *Adv. Sci.*, DOI: 10.1002/advs.202104561

Cyanine-Doped Lanthanide Metal-Organic Frameworks for Near-Infrared II Bioimaging

Tao Liang, Zhi Guo, Yifan He, Yanying Wang, Chunya Li, Zhen Li,* and Zhihong Liu**

Supporting Information

Cyanine-Doped Lanthanide Metal-Organic Frameworks for Near-Infrared II Bioimaging

Tao Liang, Zhi Guo, Yifan He, Yanying Wang, Chunya Li, Zhen Li,* and Zhihong Liu**

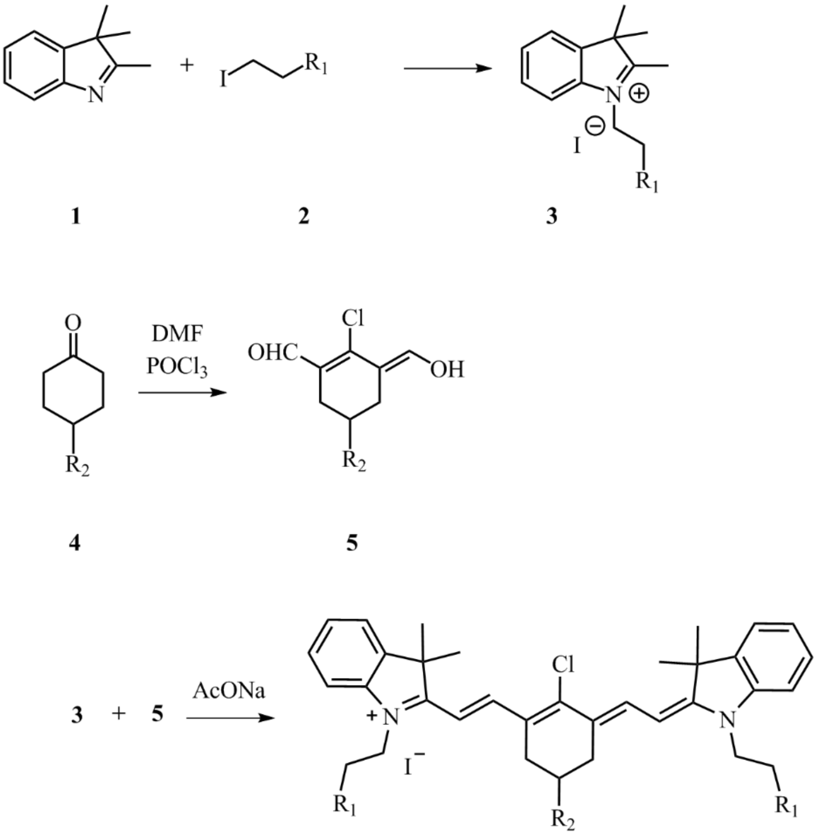

IR-3C: R_1=R_2=COOH ; IR-2C: R_1=COOH, R_2=H ; IR-0C: R_1=R_2=H

Scheme S1. Synthetic route of IR-3C, IR-2C and IR-0C.

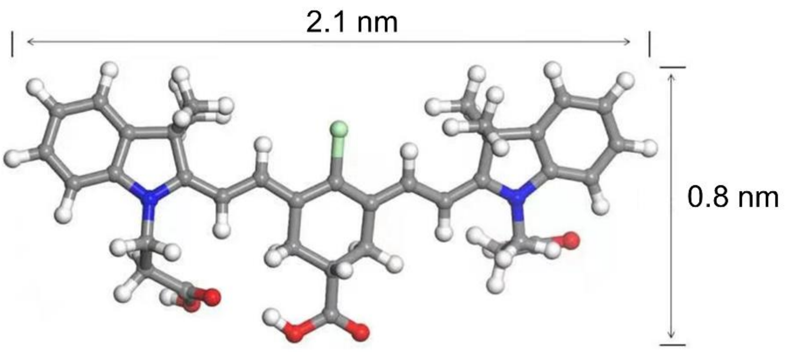

Figure S1. Chemical structure and size of IR-3C. The structure was optimized by the Gaussian 16 software based on the density functional theory (DFT) using the ωB97XD functional and the 6-31G(d,p) basis set.

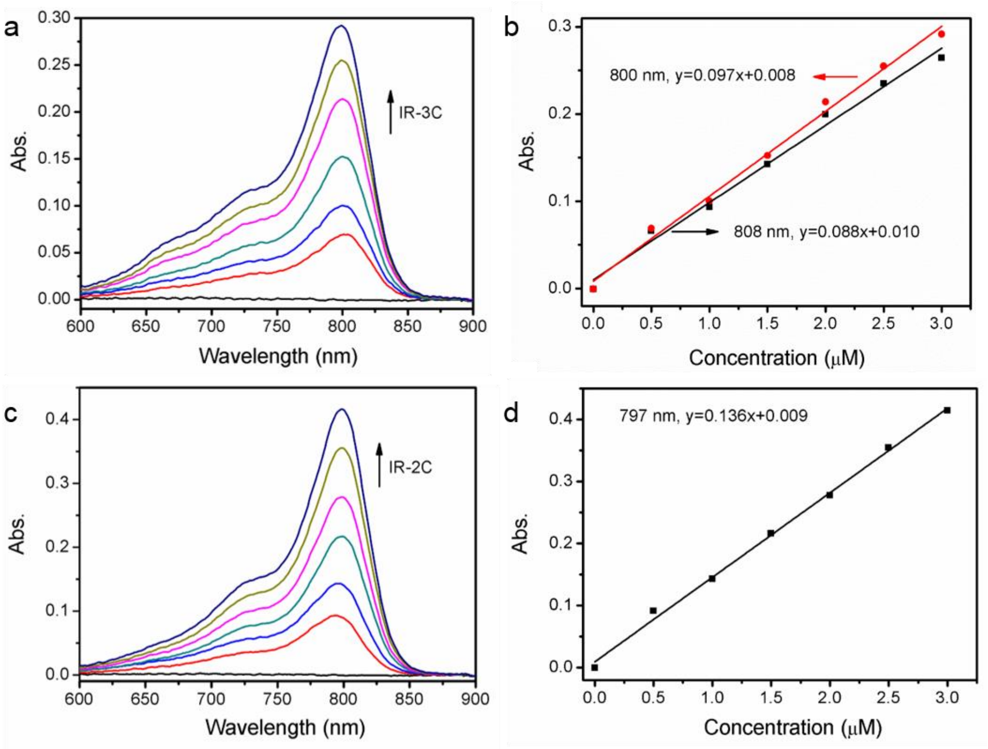

Figure S2. Absorption spectra (a, c) and the corresponding absorbance at the peaks (b, d) of IR-3C (a, b) and IR-2C (c, d) with various concentrations in DMSO.

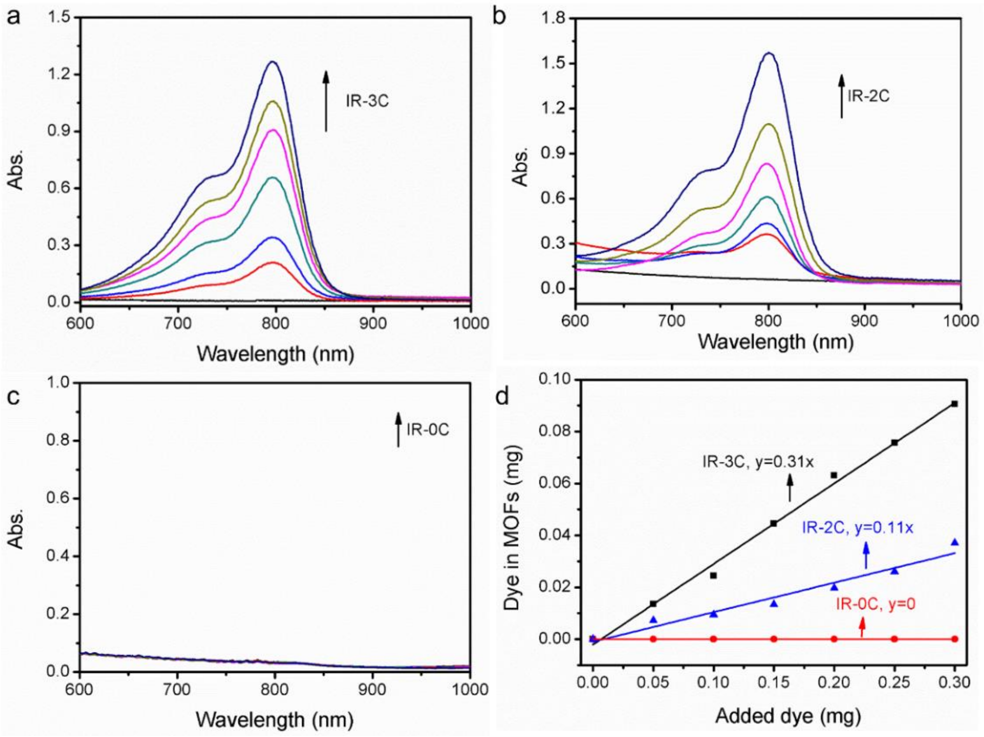

Figure S3. Absorption spectra of synthesized Er-BTC after doping with various contents of IR-3C (a), IR-2C (b) and IR-0C (c). (d) The relationship between the content of dyes successfully doped in Er-BTC and the content of added dye for synthesizing of Er-BTC. The concentrations of the obtained material for absorption spectra measurement were 0.5 mg/mL in (a) and 1 mg/mL in (b, c).

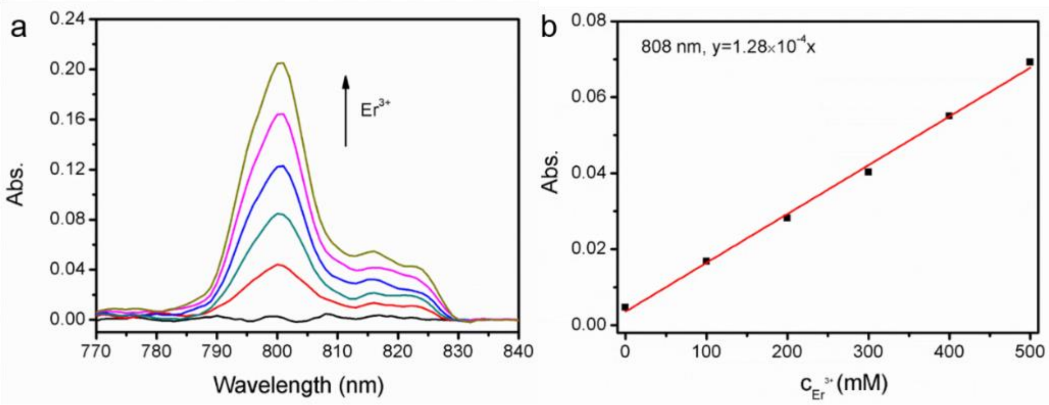

Figure S4. Absorption spectra (a) and the corresponding absorbance at 808 nm (b) of $ErCl_3$• $6H_2O$ in DMSO with various concentrations.

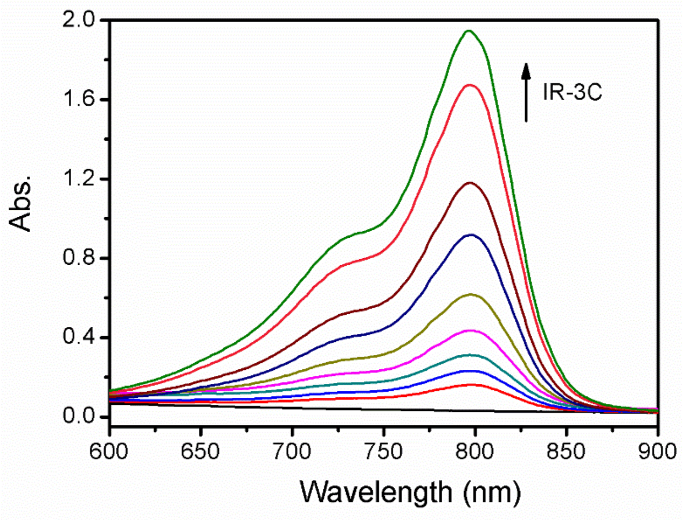

Figure S5. Absorption spectra of Er-BTC-IR doped with various contents of IR-3C.

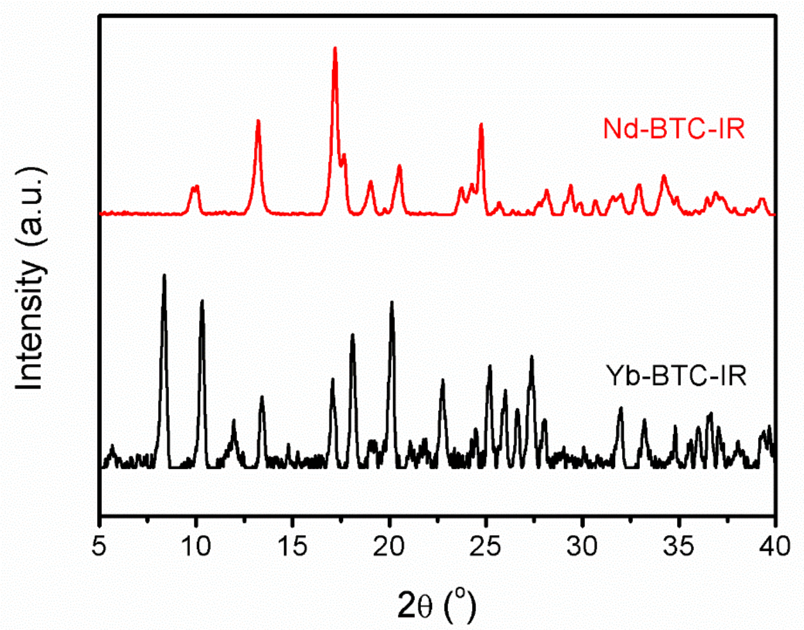

Figure S6. XRD patterns of Yb-BTC-IR and Nd-BTC-IR.

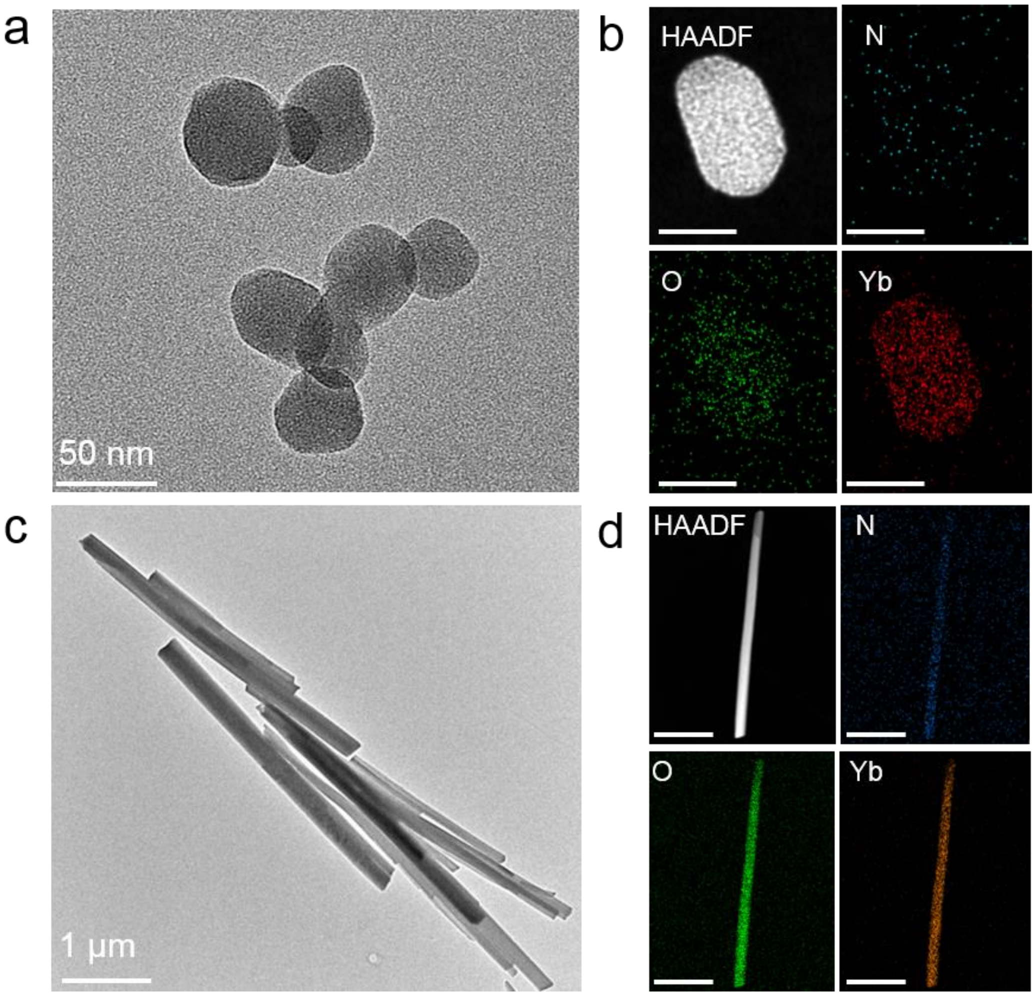

Figure S7. TEM image (a), HAADF and corresponding elemental mapping (b) of Yb-BTC-IR. TEM image (c), HAADF and corresponding elemental mapping (d) of Nd-BTC-IR. Scale bar: 25 nm in (b) and 500 nm in (d).

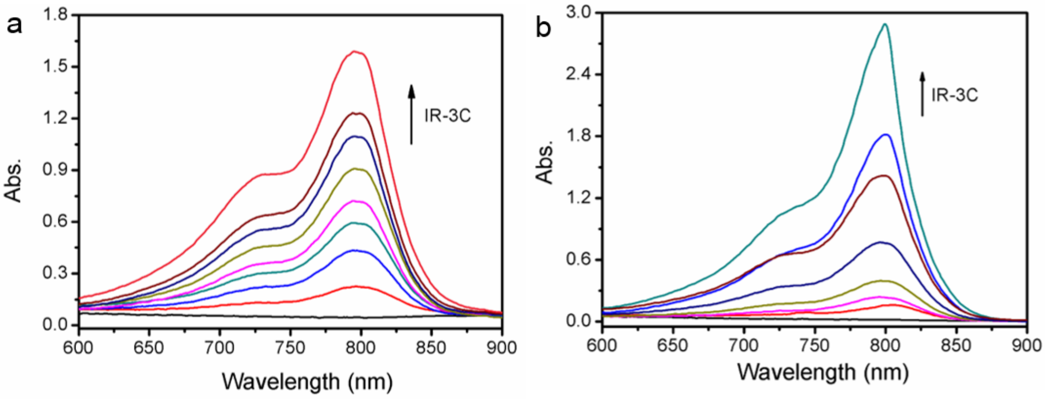

Figure S8. Absorption spectra of Yb-BTC-IR (a) and Nd-BTC-IR (b) doped with various contents of IR-3C.

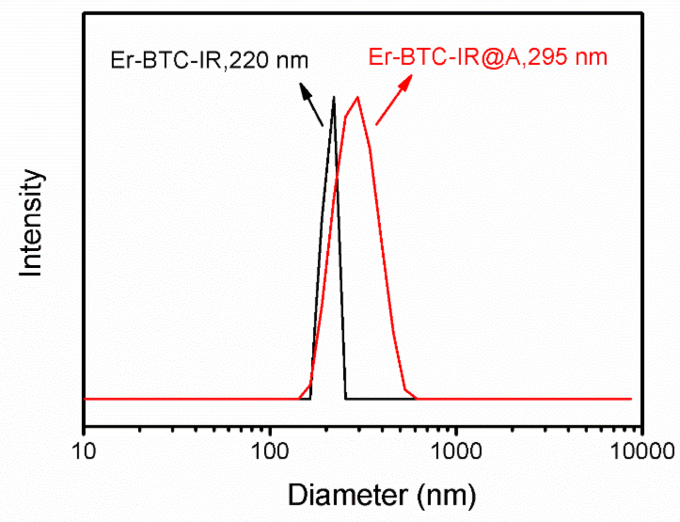

Figure S9. Hydrodynamic diameters of Er-BTC-IR and Er-BTC-IR@A.

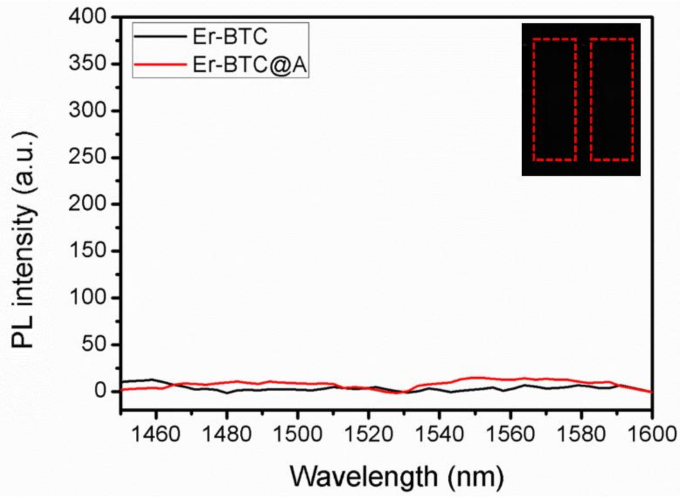

Figure S10. NIR-II luminescence spectra and photographs of Er-BTC and Er-BTC@A under excitation of 808-nm laser. The photographs were obtained by *in vivo* NIR-II imaging system equipped with an 808-nm laser and 1300 nm LP optical filter. The left one is Er-BTC and the right one is Er-BTC@A.

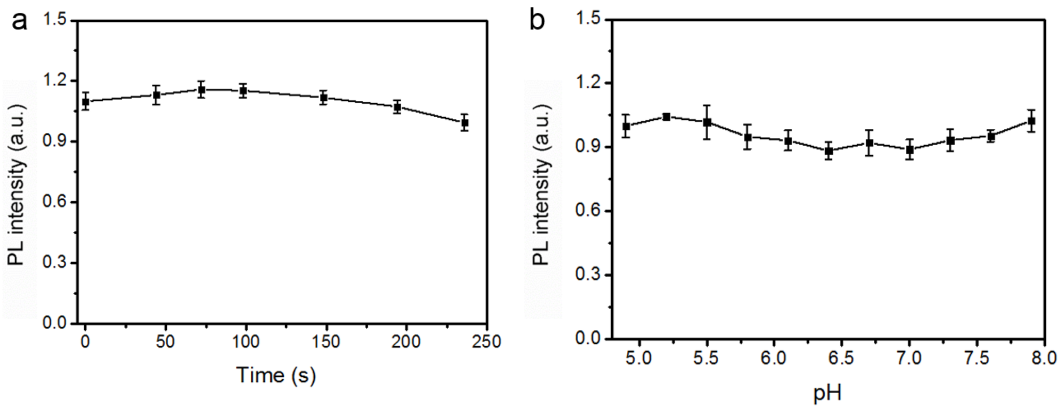

Figure S11. NIR-II luminescence intensities of Er-BTC-IR@A after irradiation at 808 nm for different time (a) and incubation for 30 min in buffer with different pH (b). Data were shown as Mean ± SD (n=3).

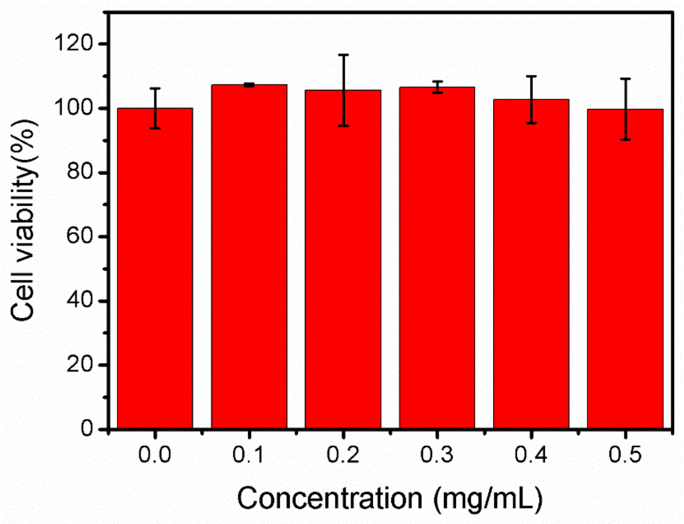

Figure S12. The viability of HeLa cells after incubation with various concentrations of Er-BTC-IR@A for 24 h. Data were shown as Mean ± SD (n=3).

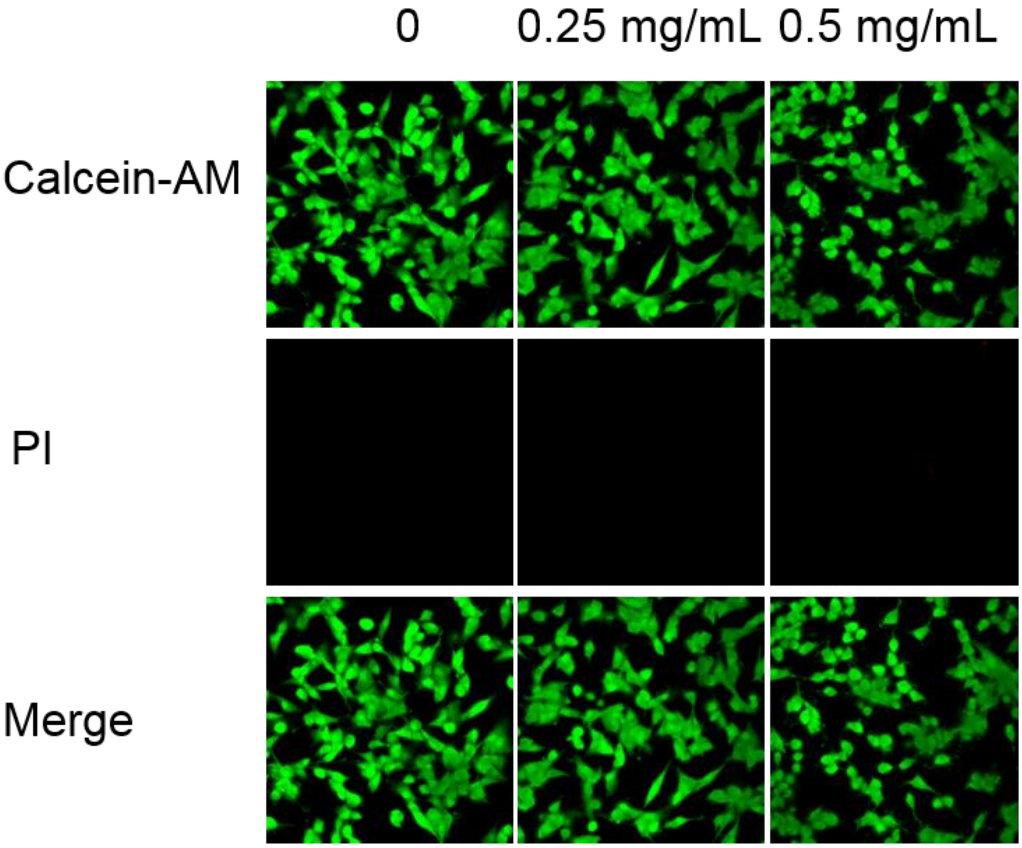

Figure S13. Confocal images of PI and Calcein-AM co-stained cells after incubation of various concentrations of Er-BTC-IR@A for 24 h.

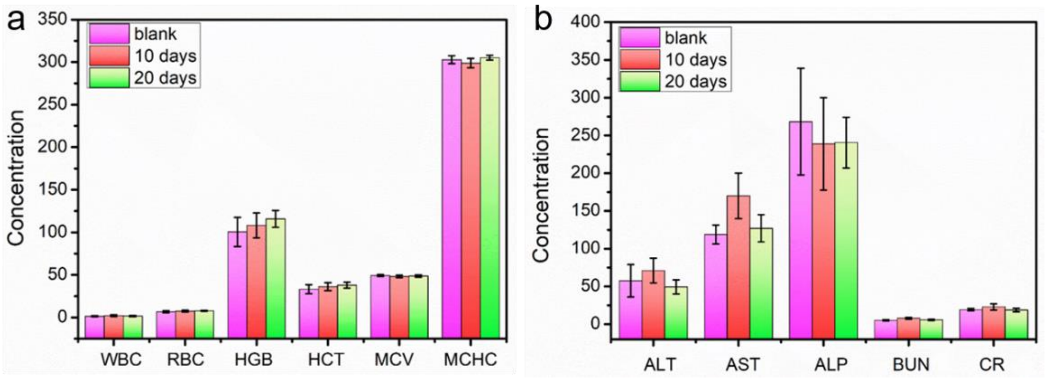

Figure S14. Blood routine (a) and serum biochemical levels (b) of Kunming mice after injection with Er-BTC-IR@A or physiological saline (blank) for various time. WBC (10^9/L), RBC (10^{12}/L), HGB (g/L), HCT (%), MCV (fL), MCHC (g/L), ALT (U/L), AST (U/L), ALP (U/L), BUN (mg/dL) and CR (U/L) represent white blood count, red blood count, hemoglobin, hematocrit, mean corpuscular volume, mean corpuscular hemoglobin concentration, alanine aminotransferase, aspartate aminotransferase, blood urea nitrogen and creatinine, respectively. Data were shown as Mean ± SD (n=5).

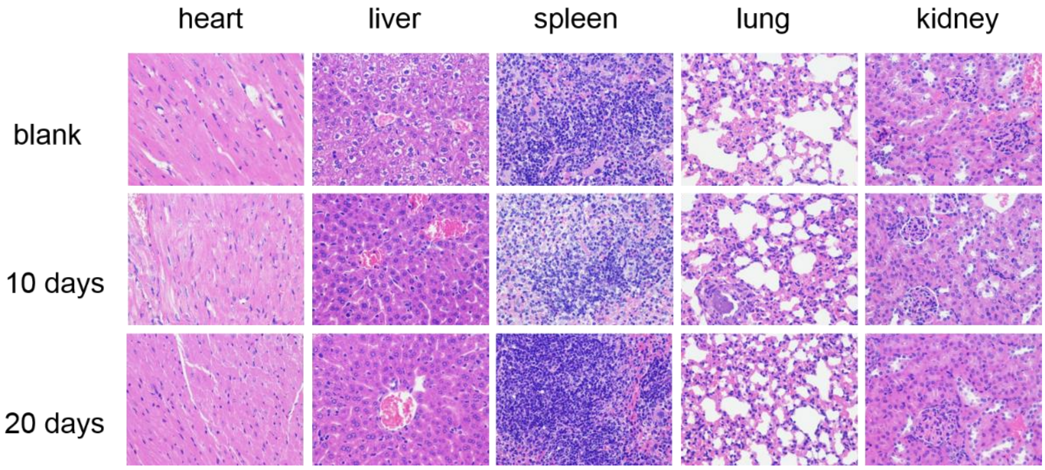

Figure S15. H&E staining of major organs of Kunming mice after injection with Er-BTC-IR@A or physiological saline (blank) for different time.

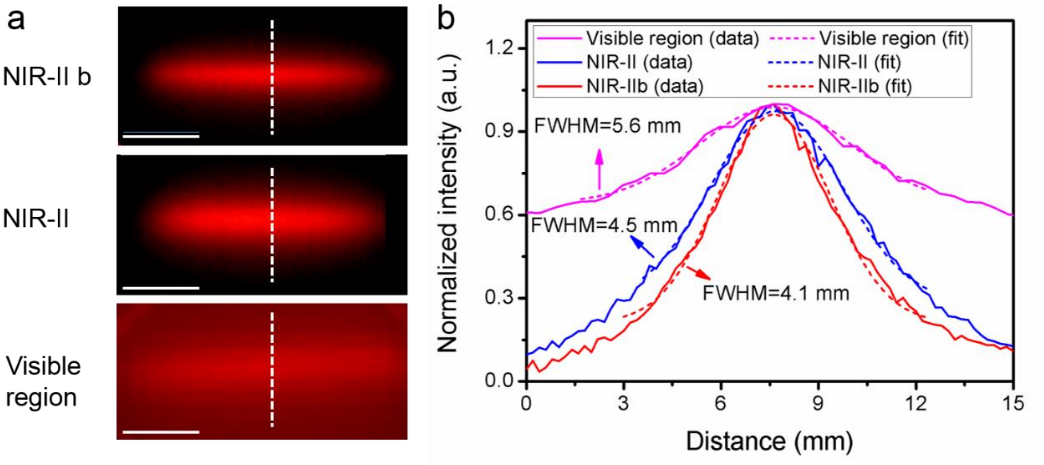

Figure S16. The luminescence images (a) and the corresponding cross-sectional intensity profiles, Gaussian functional fitting lines and FWHM values (b). Luminophors were filled into a same capillary and then covered by 1% intralipid with thickness of 4 mm. Er-BTC-IR and Nd-BTC-IR were excited by 808-nm laser equipped with a corresponding LP filter to obtained NIR-IIb and NIR-II image, respectively. Commercial organic dye Cy5 was used to obtain the image in visible region under excitation of 620-nm light (emission in the range from 640 nm to 660 nm was collected).

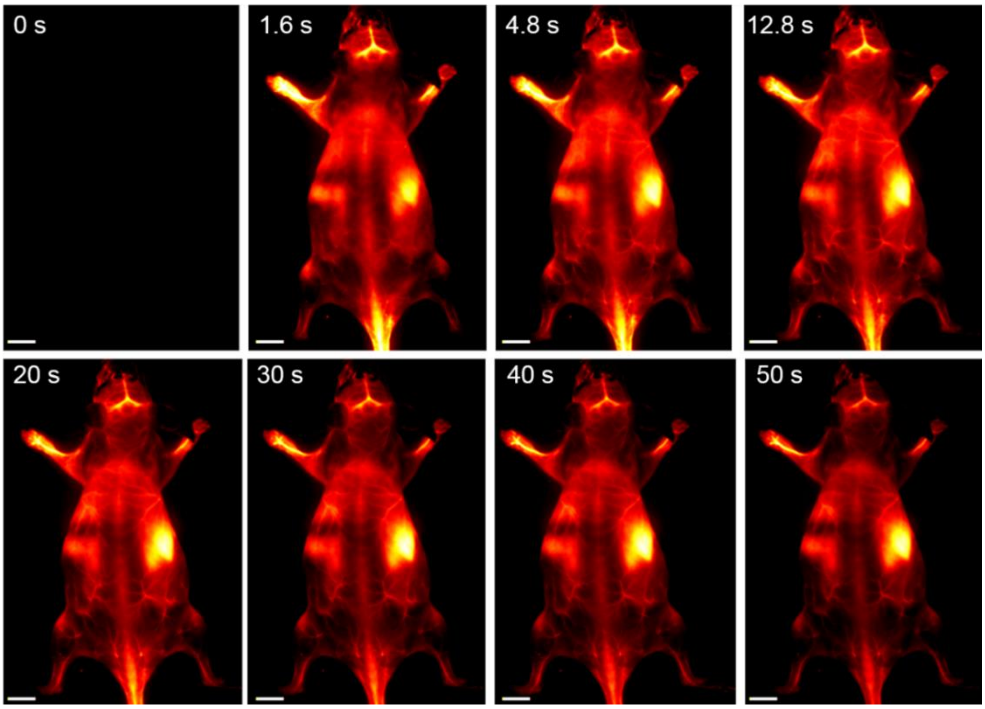

Figure S17. NIR-IIb luminescence images of Kunming mouse after injection with Er-BTC-IR@A at different time points. Scar bar: 1 cm.

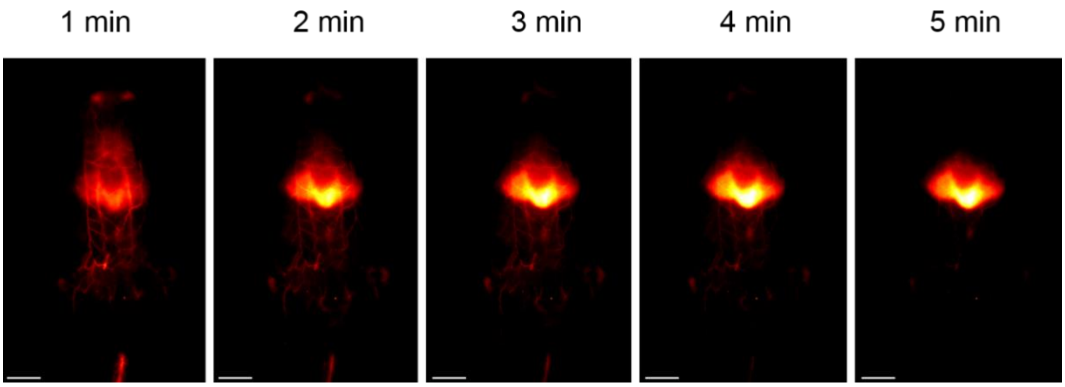

Figure S18. NIR-IIb luminescence images of Kunming mouse after injection with Er-BTC-IR@A at different time points (abdomen). Scar bar: 1 cm.

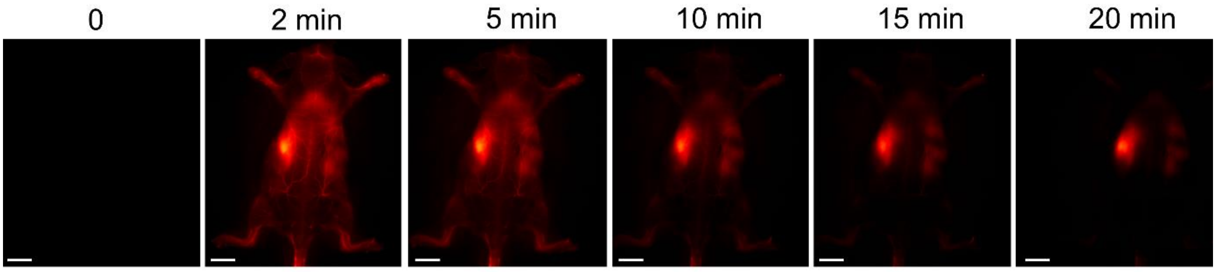

Figure S19. NIR-IIb luminescence images of Kunming mouse after injection with Er-BTC-IR@A at different time points (backside). Scar bar: 1 cm.

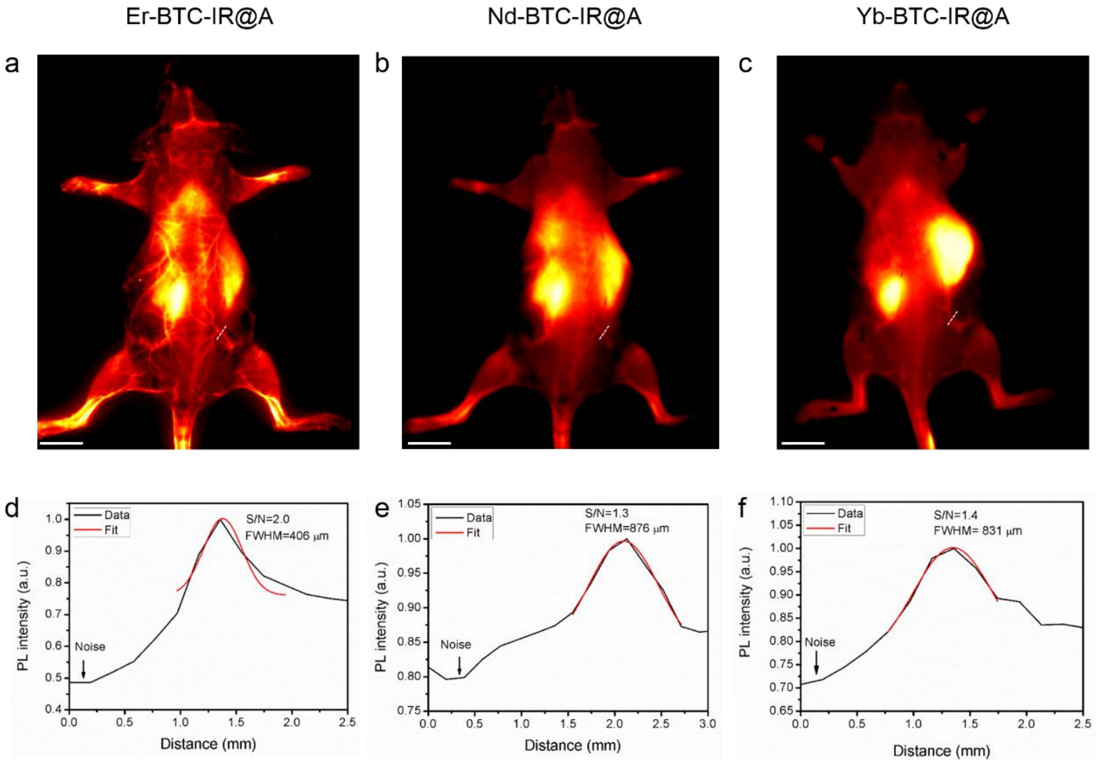

Figure S20. NIR imaging of Kunming mouse (a-c) and corresponding cross-sectional intensity profiles, Gaussian functional fitting lines, FWHM values and signal-to-noise ratio (S/N) along the dotted white lines in a-c (d-f) after injection of Er-BTC-IR@A (>1300 nm, a,d), Nd-BTC-IR@A (>1000nm, b,e) and Yb-BTC-IR@A (>1000 nm, c,f). Scar bar: 1 cm.

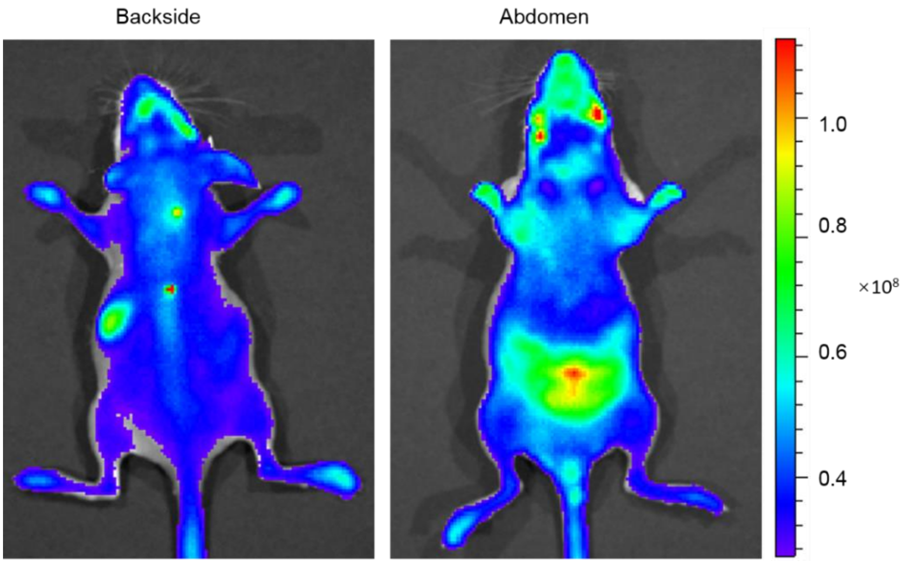

Figure S21. Fluorescence imaging of Kunming mouse in the visible region after injection with Cy5. The excitation and emission wavelength was passed through a 605 nm BP filter and 660 nm BP filter.

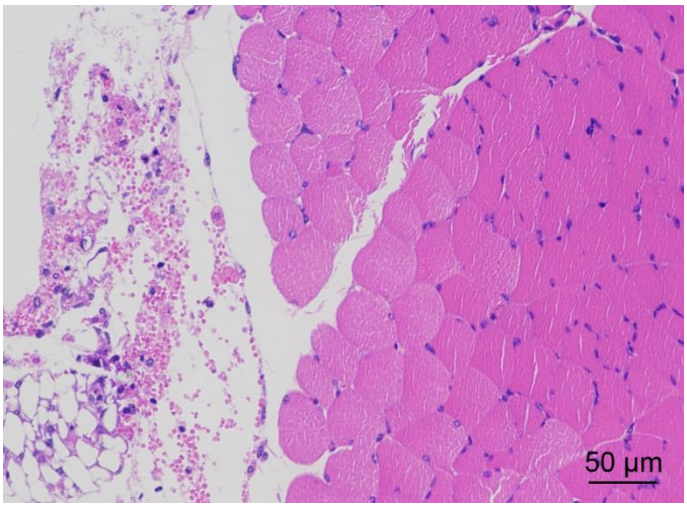

Figure S22. H&E stain of the hindlimb after injection with physiological saline for 4 h.

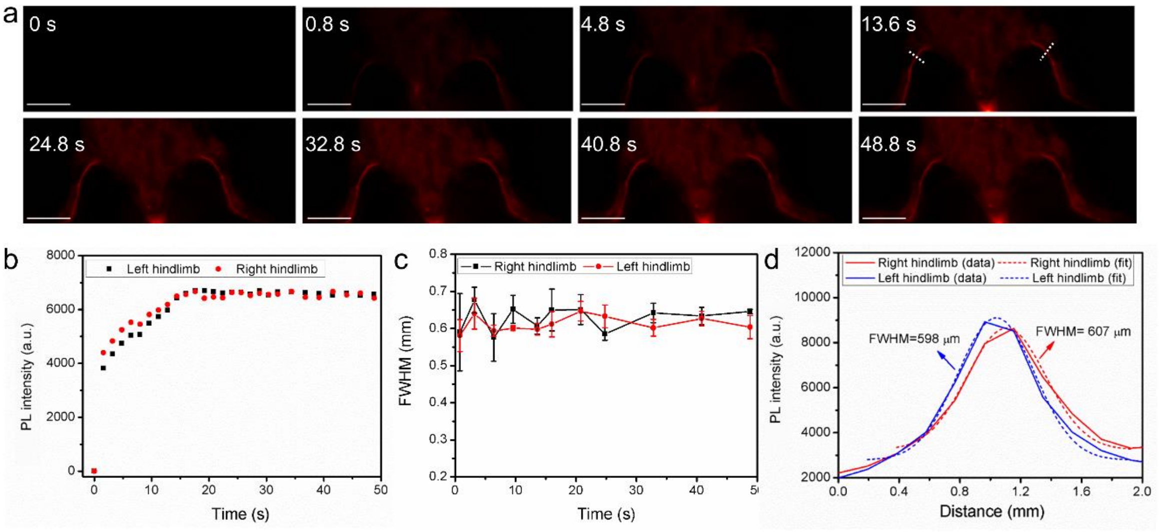

Figure S23. (a) NIR-IIb images of normal Kunming mice. The time course of fluorescence intensity (b) and FWHM (c) of vessels marked by white dotted lines in (a). Data were shown as Mean ± SD of 3 animals. (d) Cross-sectional intensity profiles, Gaussian functional fitting lines and FWHM values at 13.6 s after injection with Er-BTC-IR@A. Scale bar: 1 cm.

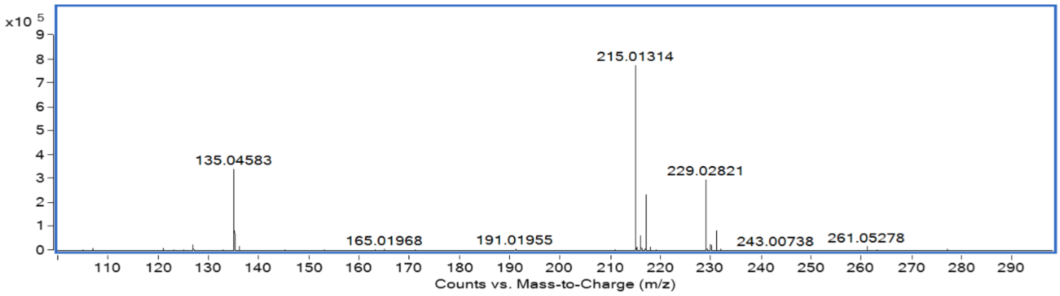

Figure S24. High resolution mass spectrum (HRMS) of compound **5**.

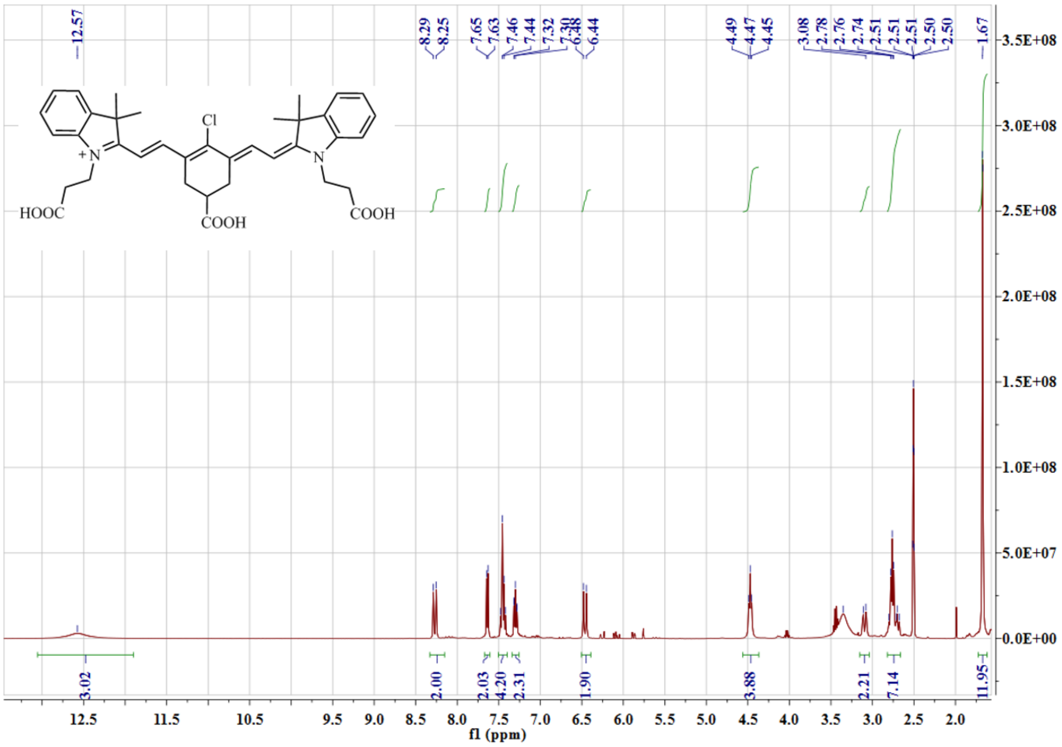

Figure S25. ^{1}H NMR spectrum of IR-3C.

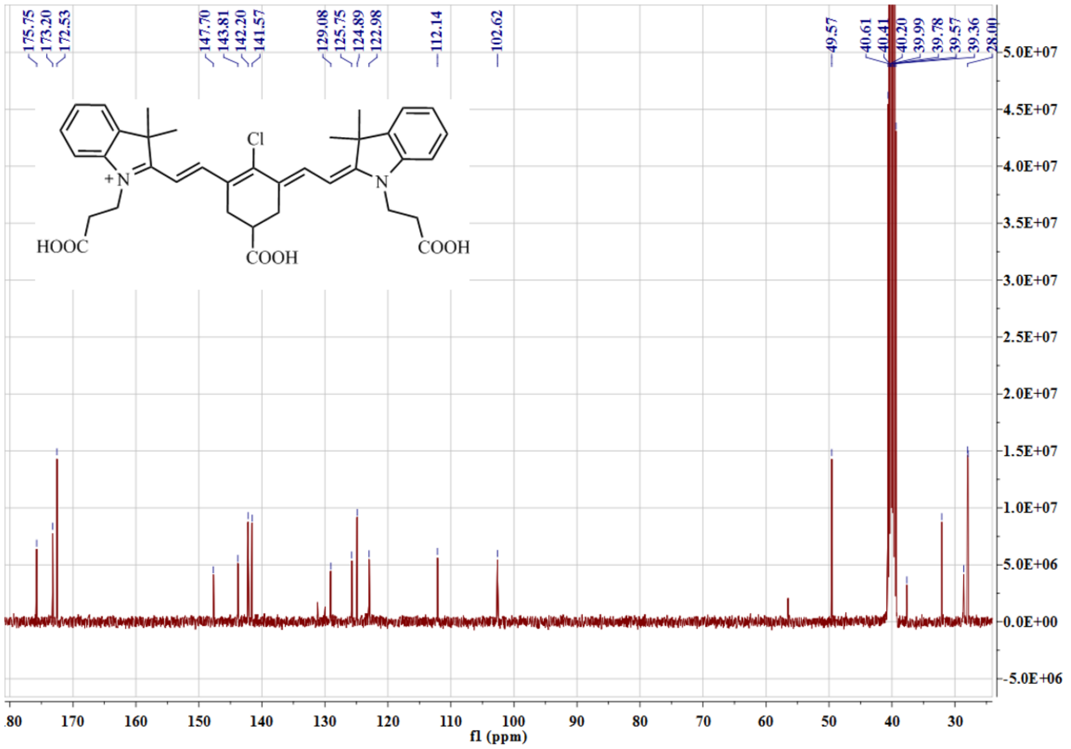

Figure S26. ^{13}C NMR spectrum of IR-3C.

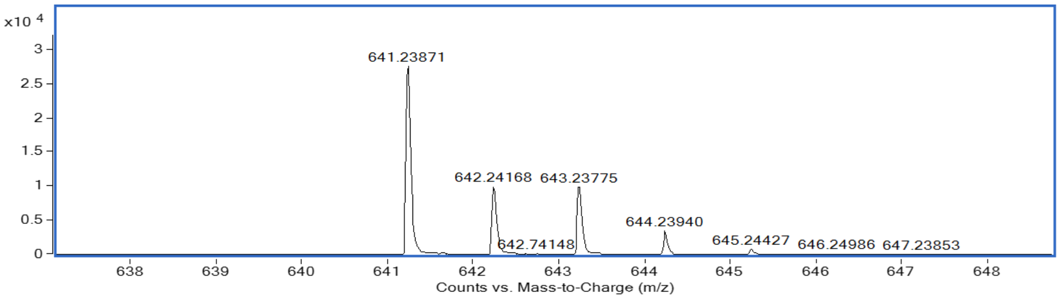

Figure S27. HRMS of IR-3C.
